# Integration of datasets from different analytical techniques to assess the impact of nutrition on human metabolome

**DOI:** 10.3389/fcimb.2012.00156

**Published:** 2012-12-11

**Authors:** Pamela Vernocchi, Lucia Vannini, Davide Gottardi, Federica Del Chierico, Diana I. Serrazanetti, Maurice Ndagijimana, Maria E. Guerzoni

**Affiliations:** ^1^Interdipartimental Centre for Industrial Research-CIRI-AGRIFOOD, Alma Mater Studiorum, University of BolognaBologna, Italy; ^2^Parasitology Unit, Department of Laboratories, Bambino Gesù Children's Hospital, IRCCSRome, Italy; ^3^Department of Agricultural and Food Sciences, Alma Mater Studiorum, University of BolognaBologna, Italy; ^4^Department of Agricultural Food and Nutritional Science, University of AlbertaEdmonton, AB, Canada

**Keywords:** metabolites, instrumental methods, gut microbiota, diet, biomarkers

## Abstract

Bacteria colonizing the human intestinal tract exhibit a high phylogenetic diversity that reflects their immense metabolic potentials. The catalytic activity of gut microbes has an important impact on gastrointestinal (GI) functions and host health. The microbial conversion of carbohydrates and other food components leads to the formation of a large number of compounds that affect the host metabolome and have beneficial or adverse effects on human health. Metabolomics is a metabolic-biology system approach focused on the metabolic responses understanding of living systems to physio-pathological stimuli by using multivariate statistical data on human body fluids obtained by different instrumental techniques. A metabolomic approach based on an analytical platform could be able to separate, detect, characterize and quantify a wide range of metabolites and its metabolic pathways. This approach has been recently applied to study the metabolic changes triggered in the gut microbiota by specific diet components and diet variations, specific diseases, probiotic and synbiotic food intake. This review describes the metabolomic data obtained by analyzing human fluids by using different techniques and particularly Gas Chromatography Mass Spectrometry Solid-phase Micro Extraction (GC-MS/SPME), Proton Nuclear Magnetic Resonance (^1^H-NMR) Spectroscopy and Fourier Transform Infrared (FTIR) Spectroscopy. This instrumental approach has a good potential in the identification and detection of specific food intake and diseases biomarkers.

## Introduction

The human intestine is home of some 100 trillion of microorganisms of at least hundreds species. The density of bacterial cells in the colon has been estimated at 10^11^–10^12^ per ml, which makes it one of the most densely populated known microbial habitats (Eckburg et al., 2005; Ley et al., 2006; Vitali et al., 2010). This microbial ecosystem serves numerous important functions for the human host, including protection against pathogens, nutrient processing, stimulation of angiogenesis, modulation of intestinal immune response, and regulation of host fat storage (Palmer et al., 2007). The composition of the adult gastrointestinal (GI) microbiota has been intensely studied, using both classical microbiology cultivation and, more recently, culture-independent, small subunit (SSU) ribosomal DNA (rDNA) sequence-based methods (Palmer et al., 2006, 2007; Huse et al., 2008; Vitali et al., 2010).

The prevention and treatment of gut-related diseases will strongly depend on understanding the mechanisms involved in the complex processes of digestion. However, the interactions between the diet, microbiota and host are largely unknown (Quigley, 2011; Del Chierico et al., 2012; Nicholson et al., 2012; Payne et al., 2012).

This review is addressed to evaluate the potential of different analytical approaches to identify and detect the molecules present in body fluids, such as blood, urine, feces, which can be considered originated by food digestion, diet/gut microbiota, and host/microbiota interactions as well as disease markers.

## Diet effect on human gut metabolome

Foods contain thousands of compounds which, upon digestion and metabolism, give rise to complex physiological reactions and possible changes in the intestinal microbiota profile resulting in a plethora of metabolites present in body fluids such as blood, urine, feces, and saliva. The number and diversity of chemical compounds in foods is amazing. It has been estimated that an omnivore diet exposes humans to more than 15,000 components, 8000 out of which are non-nutrients such as dietary fiber, antioxidants, prebiotics, and probiotics (Wishart, 2008).

An individual food system such as milk contains more than 200 different oligosaccharides, and the edible plants metabolome consist of more than 10,000 detectable compounds and 800 non-nutrient phytochemicals (Wishart, 2008). These molecules and their accumulation in body fluids, and in particular in urine, can be regarded as a repository of metabolites in which any nutrient or non-nutrient that is not needed or present in excess is found. Several food-specific biomarkers, e.g., related to wine, black tea, coffee, fruit, and vegetables, have been identified in urine and blood samples (Spencer et al., 2008; Wishart, 2008). Other compounds occurring in different body fluids, namely blood, can be regarded as biomarkers of physiological response to foods. In particular, they are products of lipid peroxidation such as 8-isoprostaglandin F2alpha, and/or oxidative stress markers such as malonaldehyde or glutathione.

Some of the diet components have a potentially direct quantitative and qualitative impact on the microbial species characterizing the gut microbiota. Their metabolic output can harbor physiologically active compounds for the human host which in turn provides a stable environment for proliferation. On the other hand the host has evolved and can use bacterial fermentation products as an energy source for the epithelial cells. Some of the fermentation products, e.g., short chain fatty acids (SCFAs), can positively affect the biochemical and physiological processes at colon level and support some important biological functions (Wong et al., 2006). However, the influence of the bacterial population composition on inter-individual differences in metabolites production and colon health status is poorly understood, except for the well-known relationships between some specific metabolites and metabolic diseases (Le Gall et al., 2011).

Metabolic profiling has a wide potential for understanding the complex interactions between components of the gut microbiota, and elucidate the cause/effects relationships associated with specific nutritional choices and the related shifts in the microbiota composition.

Particularly challenging is the metabolic signatures identification of many phenotypes and their linkage with nutritional choices. In fact, the whole set of metabolites which can be detected in body fluids, and which characterizes the metabolic phenotype, named “metabotype” (Waldram et al., 2009), of an individual, is affected by various intrinsic and extrinsic factors including environment, drugs, diets, lifestyle, and genetics. It should be also considered that the content of non-nutrients in diets is higher than that of nutrients. Such non-nutrients can exert a significant effect on metabolomic profiles thus resulting in several compounds that can be used as biomarkers to trace the food origin.

## Host-microbiome metabolic interactions and cell-cell communication

Mammalian–microbial symbiosis can play a role in the etiology and development of several diseases, e.g., insulin resistance, Crohn's disease (Marchesi et al., 2007; Kinross et al., 2011), irritable bowel syndrome (IBS) (Martin et al., 2006; Sartor, 2008), food allergies, gastritis and peptic ulcers, obesity, cardiovascular disease, and GI cancers (Martin et al., 2011). Activities of gut microbiota can be highly specific, and it has been reported that the establishment of Bifidobacteria is important for the development of the immune system and management of gut functions (Ouwehand, 2007). As the microbiome strongly interacts with the host to determine the metabotype, which influences outcomes of drug interventions, the knowledge of these interactions can provide personalized healthcare solutions (Martin et al., 2011). Also the diet has a key role in the gut microbiota modulation and shaping and, as a consequence, the different foods or their ingredients play a crucial role in the microbes selection and in a metabolic signaling network construction.

**Figure 1:**
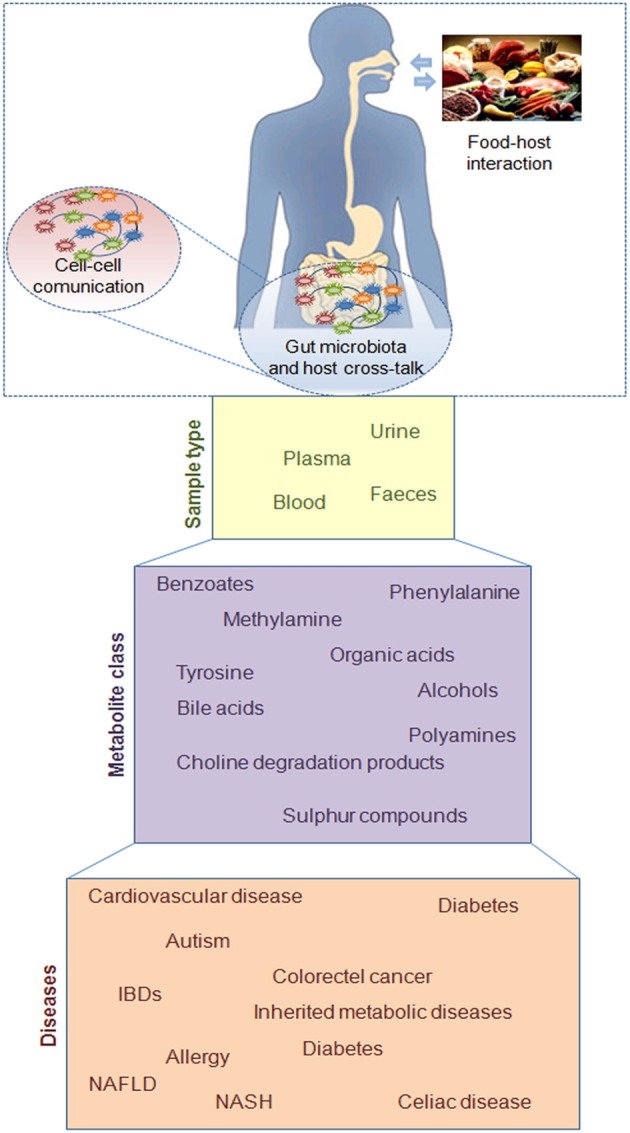
**Schematic representation of diet, microbes, and host interaction at gut level.** The chemical dialogue via low molecular weight metabolites, peptides, and proteins between cell-cell and host-microbes leads to the metabolite production in different body fluids which could be considered as disease biomarkers.

The host and its gut microbiota coproduce a large array of small molecules during the conversion of food and xenobiotics (compounds of non-host origin that enter the gut with the diet or are produced by microbiota), many of which play critical roles in shuttling information between host cells and the host's microbial symbionts. This chemical dialog includes signaling via low molecular weight metabolites, peptides, and proteins or may take place indirectly through immune-mediated pathways (Nicholson et al., 2012). The metabolites production by microbes may influence host health status and their detection in different body fluids can be considered as dysbiosis or disease biomarkers (Holmes et al., 2011; Nicholson et al., 2012) (Figure 1).

Different regions of the human GI tract vary in terms of composition of the indigenous microbiota (Gordon, 2012). For each compartment of the GI tract, a chemical dialog exists among different microbial species, e.g., direct substrate provision in the microbial food web, quorum sensing, contact-dependent signaling, and potentially gastro-transmitters (Ryan et al., 2009) as well as between microbial symbionts and host cells (Li et al., 2008).

Metabolites are the products and by-products of the many intricate biosynthetic and catabolic pathways existing in all living systems. Biological systems that exist at steady state maintain approximately constant concentrations of metabolic intermediates (Martin et al., 2011).

Fecal extracts are the most common material which can help to decipher the complex metabolic interplay between mammals and their intestinal ecosystems, and metabolic profiles-related to SCFAs, amino acids, organic acids, nucleosides and nucleotides, polyamines, phenolic compounds, bile acids, and lipid components can yield information on a range of gut diseases (Martin et al., 2011).

As described by Grider and Piland (2007), SCFAs stimulate intestinal transit, and at physiological concentrations they induce an 8–10-fold increase in serotonin release in an *in vitro* colonic mucosal system. Moreover, Musso et al. (2011) showed that SCFAs are clearly one of the most important gut microbiota products and affect a range of host processes including energy utilization, host-microbe signaling, and control of colonic pH with consequent effects on microbiota composition, gut motility, and epithelial cells proliferation.

The monitoring of the fecal metabolome also may unravel diagnostic information for inflammatory bowel diseases (IBDs), including Crohn's disease and ulcerative colitis (UC), *Hirschsprung*'*s* disease, celiac disease, allergy etc (Martin et al., 2011). Metabolomics has many potential applications. It supports functional genomics studies, systems biology, pharmacology, toxicology, and nutrigenomics. An approach called discovery metabolite profiling (DMP) is used to analyze all metabolites generated by a particular enzyme, providing a link between proteome and metabolome.

## Analytical techniques to detect metabolites

Metabonomics represents a well-recognized metabolic system approach that involves the study of multivariate metabolic responses of complex cellular organisms to different stimuli (Nicholson et al., 1999).

Ideally, it could be based on this sequence: samples collection (e.g., urine, blood, or some other body fluids), scanning them in a machine and finding a profile of tens or hundreds of chemicals that can predict whether an individual is on the road to a disease, or likely to experience side-effects from a particular drug (Pearson, 2007). However, this vision presents some drawbacks. The first complication is that one person's profile of metabolites is likely to be dramatically different from another's, and each may fluctuate markedly depending on different aspects, for example the lifestyle, the diseases, the diet, the nutrition etc (Pearson, 2007).

The detection and identification of hundreds metabolites can offer deep insights on the influence of lifestyle and dietary factors in relation to specific diseases. The recent rapid development of a range of analytical platforms, including gas chromatography (GC), liquid chromatography (LC), high pressure LC (HPLC), ultra pressure LC (UPLC) coupled to mass spectrometry (MS), capillary electrophoresis (CE) coupled to MS, Fourier Transform Infrared (FTIR) spectroscopy, and nuclear magnetic resonance (NMR) [e.g., proton (^1^H)-NMR] spectroscopy, can enable to separate, detect, characterize, and quantify such metabolites and related metabolic pathways (Zhang et al., 2011). Metabolomics focuses on the complex interactions of system components and highlights the whole system rather than the individual parts, providing a distinct perspective on cellular homeostasis (Liu et al., 2010). Even if nowadays NMR, GC-MS, LC-MS are the prevalent techniques used, none of them is a perfect technique that can meet the requirements of metabolomics for measuring all metabolites. MS-based metabolomics offers high selectivity and sensitivity for the identification and quantification of metabolites, and its combination with advanced and high-throughput separation techniques can reduce the complexity of metabolite separation (Zhang et al., 2011) (Table 1).

**Table 1:** **Common analytical techniques used in metabolomics**.

**Analytical method**	**Advantages**	**Disadvantages**	**Comments**
NMR	• Rapid analysis	• Low sensitivity	• **Chemical consideration:** gives detailed strucutural information, particularly using 2-D-NMR of isolated metabolites
	• High resolution	• Convoluted spectra	• **Chemical bias:** these methods have little chemical bias and can be used directly on the sample
	• No derivatization method	• More than one peak per component	• **Speed:** few minutes to hours. Depends on the strength of the magnet, sensitivity can be improved by magic angle spinning
	• Non-destructive	• Libraries of limited use due to complex matrix	
GC-MS	• Sensitive	• Slow	• **Chemical consideration:** on its own will not generally lead to metabolite identification. However, coupled with MS and NMR is very powerful for analyte identification
	• Robust	• Often requires derivaization	• **Chemical bias:** solvent extraction bias: non-polar vs. polar analytes. Need for chemical derivatization
	• Large linear range	• Many analytes thermally-unstable or too large for analysis	• **Speed:** very useful for separation, but typically take 10–30 min
	• Large commercial and public libraries		
LC-MS	• No derivatization required (usually)	• Slow	• **Chemical consideration:** on its own will not generally lead to metabolite identification. However, coupled with MS and NMR is very powerful for analyte identification
	• Many modes of separation available	• Limited commercial libraries	• **Chemical bias:** solvent bias means it is usually more applicable to polar compounds
			• **Speed:** very useful for separation, but typically take 10–30 min
	• Large sample capacity		
FT-IR	• Rapid analysis	• Extremely convoluted spectra	• **Chemical consideration:** provide limited structural information, but useful for identification of functional groups
	• Complete fingerprint of sample chemical composition	• More than one peak per component	• **Chemical bias:** these methods have little chemical bias and can be used directly on the sample
		• Metabolite identification nearly impossible	• **Speed:** 10–60 s
	• No derivatization needed	• Requires samples drying	

NMR-based metabolomics is able to provide a “holistic view” of the metabolites under certain conditions, and thus is well-suited and advantageous for metabolomic studies (Wu et al., 2010). In particular, it is becoming a useful tool in the study of body fluids and for a non-invasive detection of metabolites and into diagnosis of the diet effects on gut microbiota or significant public health problems (Martin et al., 2012).

Usually, the measurement of the gut microbial metabolism is confined to fecal samples, which is typically limited because of the elevated colonic absorption of bacterial metabolites. The Proton Nuclear Magnetic Resonance (^1^H-NMR) monitoring of the human fecal metabolome also may unravel diagnostic information for IBD including UC (Marchesi et al., 2007). Saric et al. (2008) evaluated the similarity and dissimilarity across different mammals namely humans, mice, and rats by using ^1^H-NMR analysis. The authors reported how human fecal extracts showed greater inter-individual variation than in rodents, reflecting the natural genetic and environmental diversity in human population.

A major source of intestinal metabolites is both host and microbial processing of dietary nutrients. As reported by Martin et al. (2010) the ^1^H-NMR analysis of feces revealed that the supplementation with probiotics significantly affects the host microbiota interaction. Probiotic supplementation of “humanized” mice, inoculated with a model of human baby microbiota, was associated with metabolic changes in the protein metabolism of *Lactobacillus paracasei* in particular with specific aminoacidic pattern.

Dumas et al. (2006) have recently applied ^1^H-NMR technique to characterize the intergenome interactions in mice with synbiotic gut microbiota as well as to monitor the gut-microbial metabolite variation in rats and to study the intricate relationships between gut microbiota and host co-metabotype associated with dietary-induced changes. Ndagijimana et al. (2009) studied, by means of the ^1^H-NMR analysis of healthy human subjects faeces, the effect of the supplementation with a synbiotic food based on *Lactobacillus acidophilus, Bifidobactrium longum*, and Fructooligosaccharides. These authors reported that the number and the extent of metabolites in faecal slurries were strongly affected by the synbiotic food consumption and gave rise to characteristic metabolic signatures. Reproducibility of ^1^H-NMR metabolic profiles generated from water and methanol extracts from human stools were assessed by Jacobs et al. (2008). On the other hand ^1^H-NMR is one of the preferred platforms also for urine and plasma analysis (Ala-Korpela, 2008). The first published study, in which a metabolomics approach on urine was described, used the ^1^H-NMR technique to monitor the effect of the inclusion of soy in the diet (Solanky et al., 2003). Urine samples have also been used to investigate responses to ingestion of chamomile tea, or other foods such as coffee, wine, and tea, evidencing that hippurate and glycine are important discriminatory metabolites (Ito et al., 2005; Wang et al., 2005).

The ability to predict the occurrence of exercise-induced ischemia in patients with suspected cardiovascular disease was investigated by ^1^H-NMR blood analysis. Barba et al. (2008) demonstrated that lactate, glucose, lipids, and long-CFAs are the main metabolites involved. Xanthine and ascorbate were proposed as possible markers of plaque formation in an artherosclerotic mouse model (Leo and Darrow, 2009) and lipoprotein subclasses can now be analyzed by a commercial ^1^H-NMR -based protocol (Jeyarajah et al., 2006; Ala-Korpela, 2007).

Even if the information provided using ^1^H-NMR is highly valuable, it is still limited due to the low resolution and sensitivity which enables the annotation and quantification of only a limited number of low molecular weight molecules (Jansson et al., 2009).

GC-MS-based metabolomics requires a high-throughput technology to handle a large volume of samples and accurate peak identification through the standard retention times and mass spectra. GC-MS has been widely used for metabolomics and can provide efficient and reproducible analysis (Zhang et al., 2011). In fact, it is also possible to obtain, simultaneously profiles of several hundred compounds including organic acids, most amino acids, sugars, sugar alcohols, aromatic amines, and FAs. GC-MS Solid-Phase Microextraction (SPME) based analysis can be considered as a very effective method for rapidly qualitatively and quantitatively analyze faecal samples most of which have not been previously reported (Garner et al., 2007). In particular, GC-MS/SPME represents a novel method to study metabolic profiles of biological samples. This approach has been used to compare neonates and adult feces (De Lacy Costello et al., 2008) and to identify volatile markers of GI diseases (Garner et al., 2007). The main metabolites identified by GC-MS/SPME belong to sulphur compounds, nitrogen compounds such as pyridine, and its derivatives, pyrazines, indoles, aldehydes, ketones, esters, alcohols, phenols, organic acids, hydrocarbons, and stress molecules such as furans and furanones. Vitali et al. (2010) investigated the impact of a synbiotic food on a human gut microbial ecology and metabolic profiles by using this technique. While no significant changes in the structure of the gut microbiota of healthy subjects was observed, the synbiotic food intake generated significant changes in some gut metabolic activities. The Canonical discriminant Analysis of Principal coordinates (CAP) of the fecal metabolic profiles showed a separation of subjects depending on synbiotic food intake. Recently, the GC-MS/SPME analysis of human feces has been also proposed as a tool to evaluate the *in vitro* effect of prebiotics and probiotics on the human microbiota (Vitali et al., 2012).

Zheng et al. (2011) applied an untargeted GC-MS-based metabonomics approach to profile bacterial metabolites in normal Wistar rats administrated with a broad spectrum β-lactam antibiotic imipenem/cilastatin sodium. In depth, metabolic phenotyping allowed the identification of 202 urinary and 223 fecal metabolites, many of which not previously reported (e.g., oligopeptides and carbohydrates), significantly related to a functional metagenome (Zheng et al., 2011). Moreover, Maccaferri et al. (2010) investigated the impact of rifaximin administration on microbial metabolic profiles by using GC-MS/SPME. At the same time this technique was also used in combination with the ^1^H-NMR technique to investigate the metabolome of 19 celiac disease children under gluten-free diet (treated celiac disease, T-CD) and 15 healthy children (HC). The metabolome of T-CD and HC children was studied using fecal and urine samples. With this approach the authors showed that the levels of volatile organic compounds and free amino acids in fecal and/or urine samples were markedly due to affected by CD (Di Cagno et al., 2011). Francavilla et al. (2012) used GC-MS/SPME to study the influence of lactose on the composition of the gut microbiota and metabolome of infants presenting with cow's milk allergy.

Moreover, MS and HPLC techniques are commonly used for compounds' characterization at structural level. In the field of metabolomics, both MS and HPLC are often combined to characterize unknown endogenous or exogenous from a complex biological matrix. LC is probably the most versatile separation method, as it allows separation of compounds of a wide range of polarity with little effort in sample preparation (compared to GC-MS) (Moco et al., 2007). Indeed, large-scale metabolomic technologies based on LC-MS are increasingly gaining attention for their use in human disease diagnosis (Courant et al., 2009).

In addition, GC-MS and LC-MS have been integrated to provide the comprehensive metabolic signature of the malnutrition in rat model and to discover differential metabolites (Wu et al., 2010).

Recently, the combination of UPLC with MS has covered a large number of polar metabolites enlarging the number of detectable analytes.

Like NMR, vibrational spectroscopies such as RAMAN and FTIR are comparable in sensibility, but the latter allows high-throughput screening and biological samples classification, and equally fits the “omics philosophy” of providing unbiased, whole-system measurements (Kell, 2004). In particular, the use of FTIR spectroscopy to monitor biochemical changes in living cells has gained considerable importance in the last decade. In fact, this technique presents the possibility to simultaneously identify various cellular biochemical targets, both *in vivo* and *in vitro* conditions, exploiting the differential infrared radiation absorption of each metabolites at specific wave number (Salman et al., 2004). Molecules and systems of biological relevance that can be detected by FTIR spectroscopy including molecules such as lipids and fatty acids, proteins, peptides, carbohydrates, nucleic acids as well as biomembranes, animal tissues, microbial cells plants, and clinical samples (Dole et al., 2011). This technique has been employed to characterize isolated biological molecules, particularly proteins, and lipids. However, more recently it has been used, with the aid of sophisticated sampling techniques such as infrared imaging, in the diagnosis of many diseases such as cervical cancer, Parkinson's disease, Alzheimer's disease, kidney stone and arthritis (Dole et al., 2011). In fact, FTIR spectroscopy can effectively provide chemical variation information about the structure and the composition of biological material at molecular level. Li et al. (2012) reported that colitis and colon cancer can be successfully identified and discern by analyzing colon biopsies through FTIR spectroscopy and chemometrics. Also, Argov et al. (2004) used FTIR microspectroscopy to distinguish IBDs from colitis-associated colon carcinomas, when pathological symptoms are similar. In particular, the study of differences in specific regions of the infrared spectra allowed the identification of the discriminating molecules, e.g., the phosphate content and RNA/DNA ratio, as different in IBD, cancer, or normal tissues. Furthermore, FTIR spectroscopy has also been used by Bright et al. (2011) to determine the efficacy of plasma homocysteine levels on vitamin supplementation in diet in hyperlipidemic patients. Human plasma samples from healthy and chronic lymphocytic leukemia patients have been analyzed by FTIR spectroscopy by Erukhimovitch et al. (2006), leading to identify of specific spectral peaks as associated with biomarkers of the disease. Cluster analysis of the selected spectra provided excellent classifications correlated completely with clinical data, showing, not with standing. Although these results are preliminary, but promising, rapid, effective, and economic technique which can assist in the disease diagnosis.

**Figure 2:**
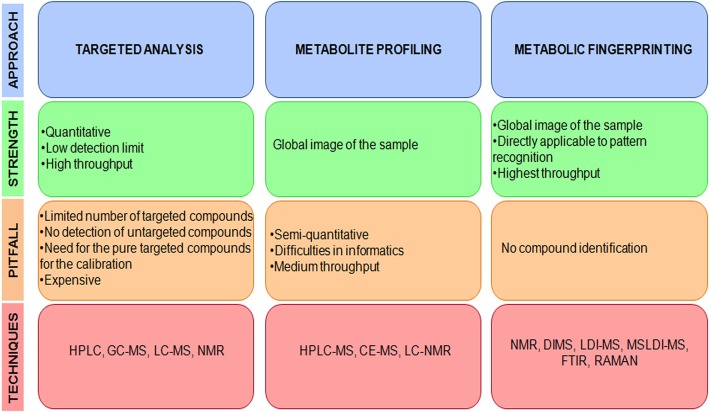
**Different approaches and respective techniques: pitfalls and strengths.** The employed technique depends on the followed approach: targeted analysis, metabolite profiling, and metabolic fingerprinting.

However, the technique selection depends on the kind of followed approach: (1) HPLC, GC-MS, LC-MS are employed in metabolite target analysis, e.g., determination and quantification of known metabolites or products of particular biochemical pathways; (2) HPLC-MS, CE-MS, LC-NMR, are used in metabolite profiling studies, e.g., larger, defined set of compounds survey and lipid analysis; (3) NMR, Direct Infusion electrospray Ionization-MS (DIMS), Laser Desorption Ionization-MS (LDI-MS), Matrix Suppressed LDI-MS (MSLDI-MS), FTIR, and Raman spectroscopy are used in metabolic fingerprinting, e.g., generation and comparison of sample metabolic profiles to identify differences (Shulaev, 2006) (Figure 2).

Furthermore, the chromatography-MS systems are considered the most favorable for the detection of large numbers of metabolites in metabolomics coupling the chromatographic metabolite separation with the sensitivity of MS detection. GC-Time of Flight (TOF)-MS, two dimensional GC coupled to TOF MS (GCxGC-TOF-MS), HPLC-MS, the analytically superior UPLC-MS and CE-MS have all been employed in mammalian metabolomic studies, either in a metabolic profiling or targeted analysis approach (Dunn et al., 2008). The GC-MS or GC-MS/SPME have been widely used in metabolomic and metabonomic studies, especially the second approach is useful because it does not require a difficult or strongly pre-analytical work flow. Garner et al. (2009) demonstrated the possibility to discriminate with GC-MS/SPME between the volatile organic compounds profile in fecal samples from preterm infants developing necrotizing enterocolitis (NEC) compared with non-NEC controls. Recent studies have suggested that the gut microbiota is involved in numerous important biochemical functions for the host, in healthy and pathological conditions (Del Chierico et al., 2012).

The extraction of valuable conclusions from the analysis of metabolomic data is important to perform the analytical measurements; in fact, there is a variety of methods that allow the instrument raw data transformation analyzed by the use of different software which provide a list of metabolites. Some parameters, such as biological variation present among individuals, sampling, sample preparation, and analytical measurement, influence the reproducibility of results, and these should be monitored as much as possible by measuring replicates, both analytical and biological. In principle, biological variance should surpass all analytical variance (Moco et al., 2007). Retention-time shifts are common in GC and more severely, LC, but only occasionally in NMR and FTIR spectra. In NMR spectra, non-reproducibility seems to be strictly related to sample preparation and hardly ever due to instrumental incoherence. Nevertheless, even in strictly controlled conditions, signal shifts may persist. For this reason, the use of signal-alignment software [e.g., MetAlign (De Vos et al., 2007), XCMS (Smith et al., 2006), and MZmine (Katajamaa et al., 2006)] has become a routine procedure for comparing chromatograms or spectra in MS applications, while HiRes is suitable in NMR (Zhao et al., 2006), transforming raw data into workable informative datasets.

## How to manage and integrate all datasets?

It is difficult to correlate specific markers with risk for disease, nutritional state or diet choices. Instead, by examining the system as a whole and exploring multiple pathways simultaneously, a greater indication of a healthy or pathological state can be acquired. Hence, this new approach, characterized by a high-throughput determination of hundreds of metabolites, leads to a torrent of data, in fact, over the years, there have been constant upgrade in the hardware and the software of these technologies to meet the demands for robustness, practicality, applicability, and efficiency of the analyses. Therefore, powerful analytical strategies in combination with advanced multivariate statistical tools are required to have a look at this new “black box” and to extract maximum relevant information and knowledge about the complex biological system (Bictash et al., 2010).

The use of single or combined analytical techniques or different kind of samples inevitably leads to face the big problem of how to manage and represent the thousands of data collected.

One of the expectations of the system biologist is that these datasets can be integrated to give a holistic picture of the state of the system, e.g., development, ageing, health, or disease, which provides insights enable a more biology fundamental understanding via unveiling network connections at molecular level (Richards et al., 2010).

Appropriate experimental design, sample numbers and statistical analyses are required to ensure generation of accurate and valid hypotheses and biological conclusions (Tseng and Wong, 2005). Powerful multivariate analyses, either supervised or unsupervised, are required to interrogate the data and define structure-related to biological system similarities or differences (Thalamuthu et al., 2006).

**Figure 3:**
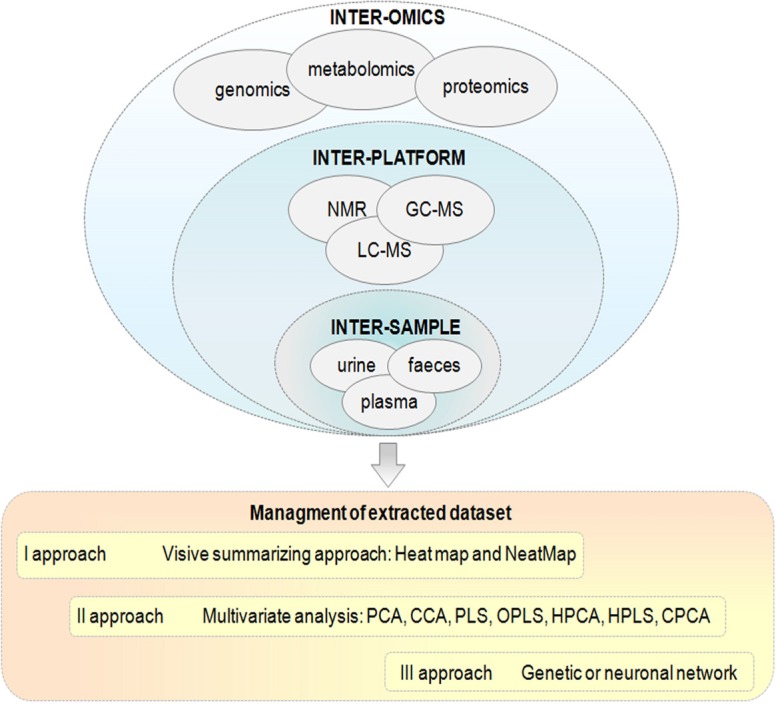
**Schematic representation of statistical data integration methods in the area of inter-omic, inter-platform, and inter-sample integration.** The management of different dataset derived from the metabolomic approaches are integrated by the most diffuse multivariate methods.

The first step for simplification of all data, but not in an arbitrary way, could be the use of Heat maps, where the relative intensity value of one peak is replaced by a small colored block (Zhou et al., 2011). Initially, it has been developed for microarray studies, but it is already used in some metabolomic works (Spitale et al., 2012). Rajaram and Oono (2010) proposed a new analysis, the so-called NeatMap as an alternative to the traditional Heat map, this offers a variety of novel plots (in 2 and 3 dimensions) to be used in conjunction with principal component analysis (PCA) and multi-dimensional scaling (MDS). Although, the NeatMap has been used so far only in genomics, it could be exploited in metabolomics as well. The second step could be the use of multivariate statistical analysis methods that can interpret different datasets. According to Richards et al. (2010) there are three possible integration strategies: (1) inter-omic, or the integration of data obtained from different -omic platforms (metabonomics, genomics, proteomics, and transcriptomics); (2) inter-platform, or the integration of data from different spectroscopic platforms (NMR, GC-MS, LC-MS etc.,); (3) inter-samples, or the integration of data obtained from different human samples (plasma, urine, tissue, and faeces) (Figure 3).

The most diffused multivariate methods are the PCA and the Partial Least Squares (PLS) (Holmes et al., 2011). In particular, PCA is widely used for multivariate of NMR or GC-MS analysis profiling data, it is based on modeling the natural variance within a dataset, which held to identify the underlying metabolite variables that contribute to that variance (Biais et al., 2009). PLS and its derivative orthogonal PLS (OPLS) have been also used for NMR profiling data. In comparison with PLS, OPLS produces models which are more clear and therefore easier to interpret, leading to a better class-resolution in a discriminant problem (Stella et al., 2006). However, when PCA and PLS are used for the interpretation of several different, but potentially connected, datasets (called “blocks”), the loading plots are usually complex due to the co-variation in the spectrum, and therefore difficult to correlate to the corresponding score plot (Janné et al., 2001). For this reason a multiblock technique must be used. The common trend of the different blocks is revealed in the “super scores” plot, where the distribution of the samples of each individual block are shown in their respective “block scores,” and similarly to classical PCA, the contribution of variables to the trend shown in the blocks scores plot is shown in their “block loadings” plot (Biais et al., 2009).

The hierarchical multiblock segmentation techniques (HPCA, HPLS) are based on new variables created from the original data by blocking the spectra into sub-spectra, and then projecting the sub-spectra by PCA. These new variables are then used in the coming PCA or PLS calculations, reducing the random and non-wanted signals from e.g., light scatter, but still conserving all systematic information in the signals. This technique gives the greatest advantage of easier interpretation of the correlation between scores and loadings (Janné et al., 2001).

Another kind of multiblock PCA used is the Consensus PCA (CPCA), it can focus the data analysis on the relation between the specified metabolites and the remaining metabolites, searches for trends that explain as much as of the variation as possible (Biais et al., 2009). CPCA was introduced as a method for comparing several blocks of descriptor variables measured on the same objects and its difference whit respect to HPCA is in the data normalization (Westerhuis et al., 1998).

Also, the multivariate analysis, such as the Canonical Correlation Analysis (CCA) and the regularized CCA are widely used to integrate datasets obtained from different source materials (Yamamoto et al., 2008). However, when the “normal” variance is greater than that explained by the process of interest, the most sophisticated genetic (Johnson et al., 2001) or neural network (Ahmed et al., 2009) based algorithms, may identify the underlying variables of significance which are not associated with the greatest variance (the “normal” condition). In these cases, such algorithm-based models should not be overfit (Biais et al., 2009).

As the holistic approach to biological systems comprehension continually evolves, the techniques used to analyze and integrate the datasets must be steadily updated to manage the problems occurring during techniques application. Hence, system biologists have a key role in the inter-pretation of the meaning of the results obtained by the datasets management.

### Conflict of interest statement

The authors declare that the research was conducted in the absence of any commercial or financial relationships that could be construed as a potential conflict of interest.
